# Mechanisms within the Parietal Cortex Correlate with the Benefits of Random Practice in Motor Adaptation

**DOI:** 10.3389/fnhum.2017.00403

**Published:** 2017-08-02

**Authors:** Benjamin Thürer, Christian Stockinger, Felix Putze, Tanja Schultz, Thorsten Stein

**Affiliations:** ^1^BioMotion Center, Institute of Sports and Sports Science, Karlsruhe Institute of Technology Karlsruhe, Germany; ^2^Neuromuscular Diagnostics, Department of Sport and Health Sciences, Technical University of Munich Munich, Germany; ^3^Cognitive Systems Lab, Faculty 3 – Computer Science, University of Bremen Bremen, Germany

**Keywords:** contextual interference, variable practice, alpha band power, electroencephalography (EEG), force field adaptation, sensorimotor learning

## Abstract

The motor learning literature shows an increased retest or transfer performance after practicing under unstable (random) conditions. This random practice effect (also known as contextual interference effect) is frequently investigated on the behavioral level and discussed in the context of mechanisms of the dorsolateral prefrontal cortex and increased cognitive efforts during movement planning. However, there is a lack of studies examining the random practice effect in motor adaptation tasks and, in general, the underlying neural processes of the random practice effect are not fully understood. We tested 24 right-handed human subjects performing a reaching task using a robotic manipulandum. Subjects learned to adapt either to a blocked or a random schedule of different force field perturbations while subjects’ electroencephalography (EEG) was recorded. The behavioral results showed a distinct random practice effect in terms of a more stabilized retest performance of the random compared to the blocked practicing group. Further analyses showed that this effect correlates with changes in the alpha band power in electrodes over parietal areas. We conclude that the random practice effect in this study is facilitated by mechanisms within the parietal cortex during movement execution which might reflect online feedback mechanisms.

## Introduction

It is widely accepted that practice under highly unstable conditions (random) compared to more stable (e.g., serial, blocked, or even constant) conditions enhances retest and transfer performance in motor sequencing tasks (Shea and Morgan, 1979; Wright et al., 2015). This random practice effect (also known as contextual interference effect) states that interference during practice is the reason for motor benefits, hence, high interference should lead to improved retention performances. This is frequently explained with the elaboration hypothesis (Magill and Hall, 1990) or reconstruction hypothesis (Lee and Magill, 1983), describing either the effect of a parallel (elaboration) or an alternating (reconstruction) motor planning of the different task conditions on motor memory consolidation (stabilization of memory over time). Nevertheless, both hypotheses have in common that retest motor benefits are explained by higher cognitive efforts during movement planning in the practice session (Li and Wright, 2000; Kantak et al., 2010). This leads to the questions if benefits of random practice truly depend on improved mechanisms of movement planning and, thus, on feedforward mechanisms and if these effects are limited to specific motor tasks or represent a general motor learning phenomenon.

It is widely accepted that movements are generated by explicit (aware) and implicit (unaware) components (Taylor et al., 2014; Huberdeau et al., 2015). Both components together enable the execution of complex motor behavior. Furthermore, if benefits of random practice are caused by cognitive effort during the planning period, it is possible that these benefits are caused by explicit but not implicit components (Kantak et al., 2010) and are processed within prefrontal brain areas (Robertson, 2009). This is in line with studies using repetitive transcranial magnetic stimulation (rTMS) showing that non-invasive brain stimulation of the dorsolateral prefrontal cortex (DLPFC) suppresses the benefit of random practice, whereas stimulation over the primary motor cortex (M1) attenuates retest performance after stable practice but not vice versa (Kantak et al., 2010). Studies using neuroimaging techniques revealed similar results showing an increased prefrontal and premotor activation when practicing under unstable conditions (Cross et al., 2007; Lin et al., 2011, 2013). Altogether, the authors of these studies conclude that memory consolidation after highly unstable practice relies on different cortical structures than consolidation after stable practice (Tanaka et al., 2010; Wright et al., 2015). This is explained by a greater involvement of prefrontal and premotor areas during movement planning (Kantak et al., 2010).

However, the above mentioned conclusion is only supported by studies which investigated the benefits of random practice by using skill and sequence learning tasks. Their observations of an increased prefrontal and premotor processing under random conditions are reasonable because these regions are strongly involved in these kinds of tasks (Pascual-Leone et al., 1996; Schwarb and Schumacher, 2009; Lin et al., 2013) and this involvement might increase under unstable task conditions. However, studies targeting at different motor tasks – in which prefrontal (Robertson, 2007) but not necessarily premotor (Hardwick et al., 2013) regions are less involved – are rare. One example would be a motor adaptation task in which subjects adapt their reaching movements to dynamic perturbations (Shadmehr and Mussa-Ivaldi, 1994) – an adaptation task which relies mainly on implicit processes (Shadmehr et al., 1998). Up to now, no study examined if the random practice effect also occurs in such a motor adaptation task and if so, whether this effect can be explained by the involvement of frontal brain regions during movement planning. An alternative explanation for a random practice effect in the motor adaptation task would be that the dynamic perturbations under random conditions are unpredictable and this uncertainty would force subjects to correct their movements during their movement execution. Such a correction would use sensory information and could be described as online feedback mechanisms (Braun et al., 2009; Yousif and Diedrichsen, 2012; Dimitriou et al., 2013). Therefore, random practice would lead to an increased integration of sensory feedback into the motor control system involving the implicit dorsal stream, which should rather affect the activity of the parietal than the prefrontal cortex (Shadmehr and Krakauer, 2008; Diedrichsen et al., 2005), and improve the correction of movements during execution. It was recently shown that such a motor control system of reaching movements is located in the posterior parietal cortex of non-human primates (Rathelot et al., 2017).

The aim of this study was to investigate if mechanisms of motor planning or motor execution, can explain the random practice effect in a motor adaptation task. Electroencephalography (EEG) was used to gain deeper insights into the neural processes of the behavioral effect. Previous EEG studies investigating memory processes in general showed that lower and higher frequency bands (theta, alpha, and higher gamma) over frontal and parietal areas are linked to memory (Canolty et al., 2006; Roux and Uhlhaas, 2014; Thürer et al., 2016). Therefore, we focused on these learning related lower and higher frequency bands in electrodes over frontal and parietal areas.

We hypothesized that a random compared to a blocked practice schedule leads to an enhanced motor memory consolidation in a dynamic motor adaptation task. Furthermore, if benefits of random practice are caused by motor execution rather than by motor planning mechanisms, this should lead to a stronger involvement of the parietal cortex during movement execution.

## Materials and Methods

### Subjects

We tested a total of 24 (age: 22 ± 2 years; six female) right-handed and healthy subjects. Handedness was assessed by the Edinburgh handedness inventory (Oldfield, 1971). All subjects provided written informed consent and had normal or corrected to normal vision. Subjects were naïve to the experimental task and the test-protocol. No subject was excluded from the analysis. The study was approved by the ethics committee of the Karlsruhe Institute of Technology.

### Experimental Apparatus and Task

**FIGURE 1:**
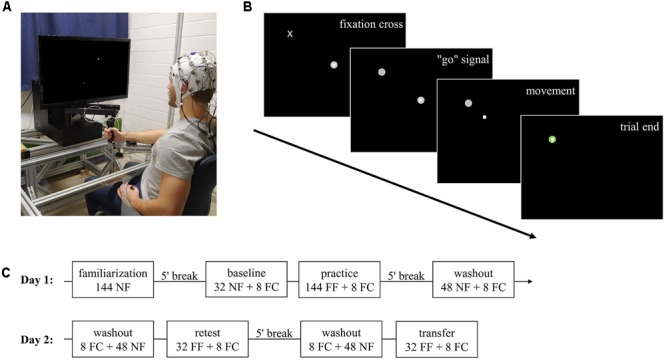
Experimental task and procedure. **(A)** Robotic manipulandum, air-sled system, and EEG. The participant gave permission to publish this figure. **(B)** Example display for one trial. Highlighting of a fixation cross which gives the subsequent “go” signal by changing to target. After subjects moved the cursor via the handle and reached the target, the trial ended and the manipulandum guided subjects’ hand back to the center position (not shown). **(C)** Experimental procedure over the two consecutive days. NF, null field; FC, force channel; FF, force field.

The experimental task was implemented by using a robotic manipulandum (Kinarm End-Point Lab, BKIN Technologies, Kingston, Canada; **Figure 1A**) which can produce forces via a handle toward subjects’ hands. Position and force at the handle of the manipulandum were recorded at a sampling rate of 1000 Hz.

Subjects were centrally positioned in front of the manipulandum and performed center-out reaching movements in the horizontal plane while grasping the handle of the manipulandum with their right hand. To prevent fatigue, subjects’ forearm was supported by an air-sled system which enabled movements with very low friction (**Figure 1A**). By handling the manipulandum, subjects controlled a cursor on a screen which was vertically located in front of the subjects. Every trial started by holding the cursor in the center target on the screen. After a fixed interval of 3.6 s, a fixation cross highlighted at the upcoming target position (**Figure 1B**). Subjects were instructed to fixate their gaze on this cross but not to start their reaching movement. This fixation cross was randomly displayed for a period of 0.8–1.5 s and then changed its shape to a circular target. Highlighting of the target served as a “go” signal. Subjects were allowed to start their reaching movements without any pressure of time (no fast reaction times required). After reaching the target, the manipulandum actively guided subjects’ hands back to the center point and, thus, provided the beginning of the next trial to a different target. In total, eight targets were arranged on a circle with a diameter of 20 cm surrounding the center target. Target order was pseudo-randomized so that in every block (one block containing eight movements) every target highlighted just once. The target order was identical across groups.

To ensure similar movement times across trials and subjects, visual feedback about the movement duration was given during the whole experiment. The feedback was displayed after finishing each trial via the target color, which became blue if the movement was too fast (<450 ms), red if it was too slow (>550 ms), and green otherwise (Thürer et al., 2016).

We implemented three types of trials: null field trials, force field trials, and force channel trials. In null field trials, the motors of the manipulandum were turned off and subjects performed movements under unperturbed conditions. The robot’s motors were turned on for the force field trials and produced a velocity-dependent curl force field in clockwise direction as follows:

$$\begin{array}{c} \left[\begin{array}{l} F_{x}\\ F_{y} \end{array}\right]=\left[\begin{array}{l} 0\;\;k\\ -k\;0 \end{array}\right]\cdot \left[\begin{array}{l} \dot{x}\\ \dot{y} \end{array}\right] \end{array}$$

where *F*_x_ and *F*_y_ are the robot-generated forces, *k* is the force field viscosity with three different gradations (*k* = 10, 15, 20 Ns/m), and ẋ and ẏ represent the horizontal components of the hand velocity. Under such force field conditions, subjects’ movements are perturbed. This typically results in an initially degraded motor performance stimulating motor learning processes (Shadmehr and Mussa-Ivaldi, 1994).

In force channel trials, the manipulandum produced a force channel from the start to the target point. Note that due to this force channel, subjects could only move directly toward the target and experienced no curl force field. This allowed the analysis of forces which subjects produced at the handle to counteract a previously learned force field task. Therefore, these forces provide a good estimation of subjects’ force field prediction and allow the measurement of subjects’ feedforward motor control (Scheidt et al., 2000; Stockinger et al., 2015). Subjects were not informed about the three different trial types.

Subsequent analyses of subjects’ performances were done using the custom made software application ManipAnalysis (Stockinger et al., 2012). To quantify the motor performance of the subjects, we calculated the absolute maximum perpendicular distance (PDmax) between subjects’ hand path and a straight line from start to target. This parameter reflects both, feedforward and feedback mechanisms. We computed the mean PDmax of the first (FT) and last (LT) 8 trials of the practice period and of the first eight trials of the retest and transfer period (practice-FT, practice-LT, retest, transfer). In addition, we were interested in subjects’ force field predictions captured using the force channel trials. In these trials, we calculated a force field compensation factor (FFcomp) by linear regression of the measured and the ideal perpendicular force profile (Joiner and Smith, 2008). As subjects do not receive error-feedback in the force channel trials, this parameter reflects mainly movement prediction by feedforward mechanisms. Clearly, we cannot rule out an additional contribution of other control mechanisms (impedance control, reflex modulation) when learning the force field. Finally, we calculated the average FFcomp over 8 consecutive force channel trials (one trial for each target direction).

### Experimental Procedure

Subjects were equally distributed in a blocked (*n* = 12; three female) and a random (*n* = 12, three female) group. The study took place on two consecutive days with 24 h between the two test sessions (**Figure 1C**).

On day 1, subjects were instructed in the behavioral task and the EEG recordings. Both groups performed 144 familiarization trials under null field conditions with two breaks of 30 s after every 48th trial to ensure that all subjects were familiarized to the task, the manipulandum, and that all subjects show the same movement speed. After a 5-min break, all subjects made a baseline measurement which started with 32 null field trials and ended with eight force channel trials. Then, subjects practiced for 144 force field trials which were divided in three parts of 48 trials with 30 s breaks between two parts. Subjects practiced the three force field gradations either in a blocked (gradation is kept constant over all trials of a part) or random (gradation changes for each trial) order with a mean force field viscosity of 15 Ns/m over the whole practice period. For the blocked group, this resulted in a Latin square design with six different gradation orders, each order practiced by two subjects. At the end of the practice period, all subjects performed eight force channel trials. Afterward, subjects had a 5-min break and performed 48 null field trials (washout) with 8 subsequent force channel trials.

On day 2, the experimental procedure was identical for both groups. First, subjects performed 8 force channel trials followed by 48 null field trials (washout). Then, all subjects performed a retest including 32 force field trials and 8 force channel trials. After another washout period of 48 trials, subjects performed 32 force field and 8 force channel trials under transfer conditions. This transfer test was identical to the retest but the manipulandum moved the subjects’ hand to the outer targets and subjects performed their reaching movements inward. Because the eight targets were equally distributed on a circle, the movement directions and the force did not differ between retest and transfer test. The only difference between the retest and transfer test was a spatial offset of 10 cm along the reaching direction. Force field viscosity for retest and transfer test was constantly set at the mean value of the practice period (15 Ns/m).

### Electroencephalography

For electroencephalography, we used the actiCHamp system with 32 active-electrodes and the BrainVision PyCorder V1.0.6 (Brain Products, Gilching, Germany). The EEG was synchronized with the manipulandum using a direct link and the data was recorded with a sampling rate of 1000 Hz. A cap with 29 EEG electrodes was used and the electrodes were placed according to the international 10-10 system (Fp1, Fp2, F7, F3, Fz, F4, F8, FC5, FC1, FC2, FC6, T7, C3, Cz, C4, T8, CP5, CP1, CP2, CP6, P7, P3, Pz, P4, P8, TP10, O1, Oz, O2). In addition, we placed three electrodes below the outer canthi of the eyes and above the nasion to record subjects’ eye movements (Schlögl et al., 2007). Electrode impedances were constantly kept below 10 kΩ. The reference electrode was placed at Cz, and electrodes were grounded to the location Fpz.

Electroencephalography data were analyzed offline using MATLAB R2015b (MathWorks Inc., Natick, MA, United States) and EEGLAB 13.5.4b (Delorme and Makeig, 2004). Raw data were high-pass filtered using a FIR filter with a cut-off frequency of 0.5 Hz. To remove 50 Hz line noise, we used the cleanline plugin for EEGLAB. Then, the data was resampled to 250 Hz and an automatic subspace reconstruction (ASR) with a “BurstCriterion” of 20 was implemented to remove bad channels and correct for movement artifacts. This step removed on average 2.8 channels (SD: 1.1) in which mostly the EOG channels were affected but in over 60% of the cases channel TP10 was also affected. Therefore, we removed channel TP10 from the whole analysis of all subjects to avoid subsequent influences on the independent component analysis (ICA). In a next step, electrodes were re-referenced to the average-reference and the signal of the channel location Cz was reconstructed and appended to the data. Then, EEG data was epoched to segments of 7 s ranging from 2 s before to 5 s after the highlighting of the fixation cross. Infomax ICA (Makeig et al., 1996) was performed with several iterations, each done on the principal components of the residual channels. In each iteration, a maximum of three components was removed if components showed distinct artifacts in the spatial, spectral, or temporal domain. On average, this procedure removed 8.4 components (SD: 3.2) of the data. Missing EEG channels due to ASR were re-calculated using spherical interpolation (except TP10).

To investigate subjects’ time-frequency power, we used complex Morlet wavelet convolution for the frequency decomposition. Therefore, 30 frequencies from 2 to 90 Hz were calculated in logarithmical space with 3 to 16 wavelet cycles changing as a function of frequency. In a next step, we increased the signal-to-noise ratio by averaging across trials. For this purpose, we computed the percentage power across all trials of the practice period (practice-ALL), of the first and last 32 trials of the practice period (practice-FT, practice-LT), and of all 32 trials of the retest and transfer period. The percentage power shows if the power of a certain time-frequency bin in- or decreases according to a fixed reference period (Pfurtscheller and Lopes da Silva, 1999). To do so, we determined a fixed reference period from 500 ms before up to the highlighting of the fixation cross. Each time-frequency bin (averaged over trials) was subtracted and divided by the mean reference period and then multiplied by 100.

According to our hypotheses, we constrained the percentage power in the time and frequency domain. In the frequency domain, motor learning processes are mostly associated with theta, alpha and gamma bands (Canolty et al., 2006; Tombini et al., 2009; Gentili et al., 2011; Perfetti et al., 2011). Especially theta and gamma bands are correlated with improvements in cognitive learning processes (Canolty et al., 2006) which makes these two frequency bands intriguing in terms of possible cognitive demands during movement planning. Therefore, we averaged the data into lower and higher frequency bands: theta (4–7 Hz), alpha (8–13 Hz), and gamma (60–80 Hz). In addition, we restricted the time domain of the percentage power to a planning (-0.4 s < *t* < 0 s) and movement (0 s < *t* < 0.4 s) window, where 0 s indicates movement onset.

### Statistics

Regarding the motor performance, we were interested in group (random, blocked) differences and interaction between group (random, blocked) and time (practice-FT, practice-LT) in the adaptation during the practice period. In addition, we were interested in the random practice effect describing an enhanced motor memory consolidation indicated by the interaction between group (random, blocked) and time (practice-LT, retest; practice-LT, transfer). Therefore, statistical comparisons were done using independent *t*-tests and mixed model ANOVAs.

To investigate effects in the EEG, we used non-parametric permutation testing on the basis of clusters (Cohen, 2014) either across the group dimension using *t*-tests or across the group and time dimensions using mixed model ANOVAs. Therefore, we computed 2d topographical head plots of the planning and movement window for every frequency band (theta, alpha, gamma) and time period (practice-ALL, practice-LT, retest, transfer). According to the hypothesis that mechanisms of motor planning facilitate the benefits of random practice, we compared groups in the planning window of the practice period. Therefore, we performed a *t*-test for every pixel of the topographical plot between groups (random, blocked) and stored the *t*- and *p*-values. The resulting maps of *p*-values were then used to create clusters of pixels below the threshold of *p* = 0.05. The *t*-values within each cluster were aggregated and stored as the observed *t*-value per cluster. Then, we shuffled the data over the group dimension, re-performed a *t*-test per pixel and stored the *t*- and *p*-values. Once more, clusters were computed on the map of *p*-values as described for the observed data. The *t*-values within each cluster were aggregated and, now, only the maximum *t*-value over all clusters is stored. This step of shuffling and re-computing was repeated 10,000 times resulting in 10,000 permutated maximum *t*-values. The 95th percentile of the permutated maximum *t*-values was defined as the threshold for significant clusters. Thus, observed clusters were defined as statistically significant if the observed *t*-value exceeded this threshold.

These cluster-based statistics were also performed across the group and time dimensions to investigate the random practice effect in the planning and movement window. Therefore, for every pixel of the topographical plot we performed a mixed model ANOVA and stored the *F*- and *p*-values of the interaction between time (practice-LT, retest OR practice-LT, transfer) and group (random, blocked). The subsequent steps were similar to the cluster-based statistics across the group dimension besides that, now, the data was shuffled over all dimensions (Edgington and Onghena, 2007).

Finally, we performed Spearman’s rank correlations between the behavioral and electrophysiological data. To do so, the difference of the PDmax and the FFcomp is computed for the retest and the last trials of the practice period (retest – practice-LT) as well as for the transfer and the last trials of the practice period (transfer – practice-LT). The same differences were computed for the ROIs alpha band power. Then, each correlation was performed between the difference on the behavioral level and the difference on the electrophysiological level.

For all statistical analyses, the level of significance was a priori set to α = 0.05 and Greenhouse–Geisser correction was used if assumption of sphericity was violated. Correction for multiple comparisons was done using false discovery rate and effect sizes were determined using Cohen’s *d* (Cohen, 1988) or partial eta squared $\eta_{p}^{2}$ (Cohen, 1988; Richardson, 2011). Statistical analyses were done using SPSS statistics 22 (IBM, Armonk, NY, United States) and MATLAB R2015b (MathWorks Inc., Natick, MA, United States).

## Results

### No Group Differences in the Motor Performance But in the Variance during Practice

As the blocked and random group performed different schedules of the force field gradations, we were interested in statistical comparisons regarding the motor performance during the practice period. Statistical results of the PDmax revealed a significant effect of time (first trials, last trials) showing that subjects improved their motor performance during practice [*F*(1,22) = 93.72, *p* < 0.001, $\eta_{p}^{2}$ = 0.81]. No significant group (random, blocked) differences were found indicating similar performances between groups [first trials: *t*(22) = 1.39, *p* = 0.179, *d* = 0.57; last trials: *t*(22) = -0.67, *p* = 0.510, *d* = -0.27]. In addition, no significant interaction effects between group (random, blocked) and time (practice-FT, practice-LT) were observed [*F*(1,22) = 2.52, *p* = 0.127, $\eta_{p}^{2}$ = 0.10]. We also checked for differences between groups in their force field prediction by examining the FFcomp in the force channel trials at the end of the practice period. The results showed no significant differences indicating no differences in the force field prediction between groups [*t*(22) = 0.08, *p* = 0.447, *d* = 0.32].

Furthermore, we performed independent *t*-tests between the variances of the motor performance (PDmax) between groups for the first, second, and third part of the practice period, each including 48 trials. The results showed a significant effect for the second [*t*(22) = -2.61, *p* = 0.016, *d* = -1.06] but not for the first and last part after correction using FDR [first: *t*(22) = -0.39, *p* = 0.696, *d* = -0.16; third: *t*(22) = -1.82, *p* = 0.083, *d* = -0.74].

Summarized, both groups increased their motor performance during the practice period but no group or interaction effects were observed. However, we found an increased variance in the motor performance for the random compared to the blocked group during practice.

### Random Practice Leads to Enhanced Retest Performance

**FIGURE 2:**
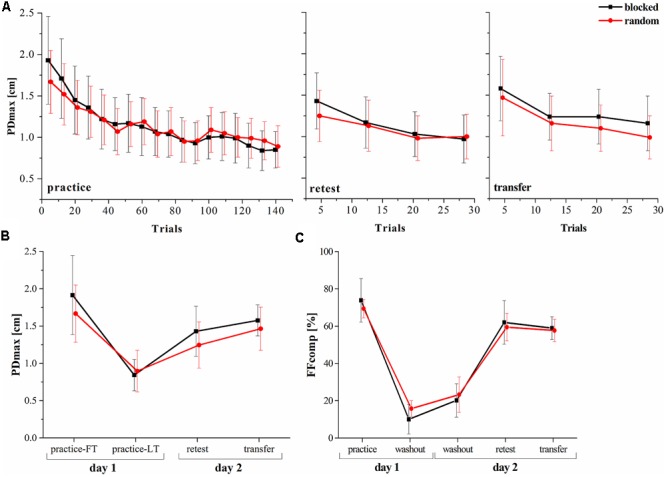
Progress of the motor performance over the two consecutive days. **(A)** Progress of the maximum perpendicular displacement (PDmax) between groups along the practice, retest and transfer tests. **(B)** PDmax between groups for specific blocks in the experiment. **(C)** Results of the force field compensation factor (FFcomp) between groups over the entire experiment (Mean ± CI_95_). FT, first trials; LT, last trials.

One of the hypotheses of this study was, that the random practice effect is detectable in a dynamic motor adaptation task. We investigated the PDmax and used a mixed model ANOVA with factors group (random, blocked) and time (practice-LT, retest). The results showed a significant time [*F*(1,22) = 74.30, *p* < 0.001, $\eta_{p}^{2}$ = 0.77] but no significant group [*F*(1,22) = 0.73, *p* = 0.404, $\eta_{p}^{2}$ = 0.03] effect. Moreover, the interaction effect was significant revealing a high effect size [*F*(1,22) = 4.82, *p* = 0.039, $\eta_{p}^{2}$ = 0.18]. This clearly shows that although both groups decreased their performances (increase of the PDmax) from the last trials of the practice period to the retest, the random practicing group tends more to a stabilization of the performance (**Figures 2A,B**).

In a next step, we used mixed model ANOVA to test for a group^∗^time effect comparing the FFcomp at the end of the practice period with the FFcomp at the very beginning of day 2 (first washout period on day 2) and detected no interaction effect [*F*(1,22) = 2.16, *p* = 0.156, $\eta_{p}^{2}$ = 0.08]. Thus, force field prediction does not explain the observed random practice effect (**Figure 2C**).

It was shown that random practice can also enhance the performance in a transfer test. Therefore, we used the PDmax to perform a mixed model ANOVA with the factors group (random, blocked) and time (practice-LT, transfer). The results showed no interaction effect [*F*(1,22) = 1.36, *p* = 0.257, $\eta_{p}^{2}$ = 0.05] and, thus, similar performances between groups over time (practice to transfer).

In summary, our behavioral data show that both groups decreased their performances from practice-LT to retest but this decrease was significantly smaller for the random group which shows the random practice effect. However, this effect is not explainable by increased force field prediction and, thus, feedforward mechanisms.

### Changes in the Alpha Band Power Coincide with the Random Practice Effect

To deal with high dimensionality of EEG data, we used cluster-based permutation tests and corrected using a maximum statistic. Statistics of the planning window during practice failed to show significant group differences in any of the analyzed frequency bands. The cluster-based statistics of the planning window testing for a group (random, blocked) and time (practice-LT, retest; practice-LT, transfer) interaction effect also did not show significant results. Thus, statistics during the planning window cannot explain the random practice effect on the behavioral level.

**FIGURE 3:**
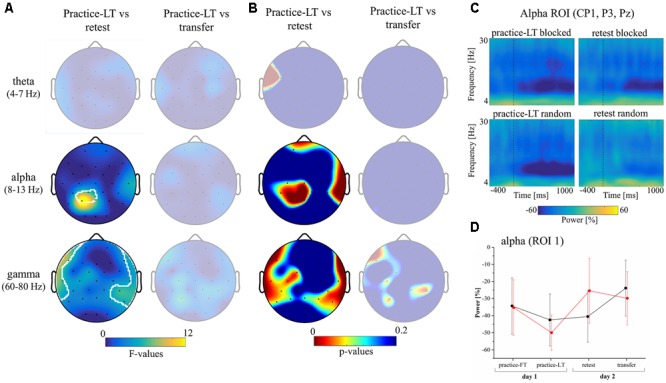
Results of the EEG data. **(A)** Topographical plots show the *F*-values of the cluster-based statistic regarding an interaction effect (group^∗^time) in the movement window for the theta, alpha, and gamma frequency bands. Columns show interaction statistics between practice-FT and retest (left) and between practice-FT and transfer (right). Significant clusters, corrected for multiple comparisons, are circled by white pixels. **(B)** Topographical plots of the *p*-values of the cluster-based statistic. **(C)** Time-frequency plots display the average alpha (ROI 1: CP1, P3, and PZ) band power during the practice-LT and retest periods from 400 ms before to 1000 ms after movement onset (dashed vertical lines indicate movement onset). **(D)** Progress of the alpha band power during movement execution over the whole experiment for both groups (Mean ± CI_95_).

Similarly, cluster-based statistics regarding the movement window showed no significant clusters regarding group differences during practice. However, statistics showed significant clusters in the alpha and gamma bands testing for interaction effects with the factors group (random, blocked) and time (practice-LT, retest; **Figures 3A,B**). No significant clusters were observed in the theta band and no significant clusters were observed comparing for a possible transfer effect (time: practice-LT, transfer; **Figure 3A**). This shows that the random practice effect which we observed on the behavioral level coincides with percentage power changes in electrodes over the parietal cortex (alpha) and temporal cortex (gamma). Regarding the gamma band, results have to be interpreted with great caution as the statistical results are comparably weak and reflect partly the interpolated activity of the outermost electrodes. In a next step, we built a region of interest (ROI) according to the significant cluster in the alpha band (CP1, P3, and PZ) and examined the time-frequency plots of the percentage power between groups (**Figure 3C**). Time-frequency plots of this ROI showed a decreased alpha band power for blocked and random groups in the practice-LT period. This decreased alpha band power was also present in the retest period but much weaker for the random group. This effect was also confirmed by the progress of the alpha band power for the ROI during the whole experiment (**Figure 3D**) which showed a significant interaction effect between group and time with a high effect size [*F*(1,22) = 12.03, *p* = 0.002, $\eta_{p}^{2}$ = 0.35].

In summary, random and blocked groups showed similar alpha power values at the beginning of the practice period but at the end of the practice period, alpha power of the random group showed a stronger decrease in power. However, the random group showed a distinct increase in alpha power from practice-LT to retest whereas alpha power in the blocked group stayed constant.

### Changes on the Behavioral Level Correlate Negatively with Changes in the Alpha Band

**FIGURE 4:**
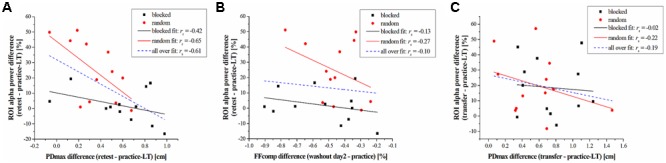
Results of the correlation analyses. **(A)** Correlation between the difference of the alpha band power (retest – practice-LT) and the PDmax (retest – practice-LT) for the blocked (black squares) and random (red circles) group. Linear fits are represented by black-solid (blocked), red-solid (random), and blue-dashed (blocked + random) lines. **(B)** Correlation between the difference of the alpha band power (retest – practice-LT) and the FFcomp (washout day 2 – practice) for the blocked and random group. **(C)** correlation between the difference of the alpha band power (transfer – practice-LT) and the PDmax (transfer – practice-LT) for the blocked and random group.

The above-mentioned results are obtained by statistical comparisons either on the behavioral or on the electrophysiological level. To investigate if there is a relationship between these results on the subject level, we used spearman correlation analyses between the alpha band power of the ROI and the PDmax or the FFcomp. The differences of these parameters between task periods (retest – practice-LT; transfer – practice-LT) were computed to take the performance change into account. Results across both groups show a significant negative correlation between the alpha band power difference and the PDmax difference from practice to the retest period (*r*_s_ = -0.61, *p* = 0.002; **Figure 4A**). This negative correlation coefficient decreases even more when taking only the random-practicing subjects into account, although it does not reveal significance after correction for multiple comparisons using FDR (*r*_s_ = -0.65, *p* = 0.026; **Figure 4A**) due to the reduced sample size. For the blocked group, the correlation coefficient was higher indicating no correlation between the alpha band power difference and the PDmax difference for the blocked group (*r*_s_ = -0.42, *p* = 0.177; **Figure 4A**). In contrast, no significant correlations are found for the FFcomp (across both groups: *r*_s_ = -0.10, *p* = 0.626; random group: *r*_s_ = -0.27, *p* = 0.404; blocked group: *r*_s_ = -0.13, *p* = 0.683; **Figure 4B**) and for the transfer period (across both groups: *r*_s_ = -0.19, *p* = 0.378; random group: *r*_s_ = -0.22, *p* = 0.499; blocked group: *r*_s_ = -0.02, *p* = 0.956; **Figure 4C**).

Summarized, EEG data showed a distinct group^∗^time interaction in the alpha band. This alpha band effect correlates with the performance changes, measured by PDmax, on the behavioral level.

## Discussion

The aim of this study was to investigate if the random practice effect is either facilitated by mechanisms of motor planning or motor execution. We used a dynamic motor adaptation task to quantify the random practice effect on the behavioral level and EEG to identify the underlying neural correlates. The behavioral results confirmed our hypothesis that random practice enhances motor memory consolidation in a motor adaptation task. All results support our hypothesis that the motor benefit is rather caused by motor execution than by motor planning mechanisms.

### The Random Practice Effect in a Motor Adaptation Task Relies on Motor Execution Mechanisms

The behavioral data show that practice under random conditions tends to an increased memory consolidation compared to blocked practice conditions. This is in line with the literature (Shea and Morgan, 1979; Wright et al., 2015) and shows that random practice leads to motor benefits even in a motor adaptation task with dynamic perturbations.

We observed the random practice effect in the PDmax, which reflects both feedforward and feedback control, but not in the FFcomp, which reflects mostly feedforward control. This shows that the random practice effect in our data is not caused by a more pronounced improvement in the force field prediction. Previous work showed that subjects adapt to the approximate mean of the task dynamics when they are exposed to unpredictable dynamic conditions (Scheidt et al., 2001). In that way, as the force field viscosity mean value across all subjects and within each group was 15 Ns/m, similar force field predictions between groups at the end of the practice period and at the retests concur with the literature. However, this force field prediction cannot entirely counteract the dynamic uncertainty during movement execution under random force field conditions. Therefore, subjects of the random group must have used either some sort of control strategy (impedance control or reflex modulation) or online feedback mechanisms to perform similar to the subjects of the blocked group (Franklin et al., 2012; Stockinger et al., 2014). Usage of an impedance strategy would lead to a minor variability in the PDmax and to a decreased reliance on force field prediction in the random group. However, since we did find an increased motor variability for the random group in the second part of the practice period and did not observe any differences in the force field prediction, the usage of online feedback mechanisms is more likely.

The assumption that online feedback mechanisms corrected during movement execution is also supported by the results of the EEG data. Results of the EEG-data failed to find significant differences between groups or a significant interaction effect during movement planning. However, EEG data showed a similar interaction effect in the alpha band power in electrodes over parietal areas during movement execution as for the behavioral data. It should be noted, that this effect also occurred when only eight trials (like for the behavioral effect) were taken for the analyses (results not shown here). The parietal cortex is a main control center for sensory feedback in the brain. Upstreaming sensory information is filtered and forwarded by the thalamus and reaches the parietal cortex where the relevant information for movement control goes along the dorsal stream and the relevant information for semantic knowledge goes along the ventral stream (Gardner and Johnson, 2013). The coincidence between the random practice effect and the alpha band power indicates such an increased feedback mechanism. This is in line with previous work showing changes in the parietal cortex activity when subjects adapted their reaching movements during movement execution to random target positions or kinematic conditions (Desmurget et al., 1999; Diedrichsen et al., 2005). Therefore, we suggest that in a dynamic motor adaptation task subjects of the random group are required to rely on online feedback mechanisms more than subjects of the blocked group.

### Random Practice Seems to Reflect a General Learning Phenomenon

Up to now, the literature on the random practice effect mostly used tasks which rely on a strong explicit component (e.g., Battig, 1972; Shea and Morgan, 1979; Kantak et al., 2010). Especially in the motor domain, previous work used skill acquisition tasks (e.g., sequencing task, sinusoid tracing, serial reaction time task) for which it is quite reasonable that random practice shows an increased involvement of prefrontal and premotor areas. As far as we know, only one study (Kim et al., 2015) used a motor adaptation task instead of a skill acquisition task to investigate the effect of random practice on retest. The amount of explicit control should be weaker in such a motor adaptation task, especially using dynamic perturbations (Shadmehr et al., 1998) and, thus, random practice should rather lead to an increased involvement of parietal than prefrontal areas as shown for uncertain kinematic conditions (Diedrichsen et al., 2005). Therefore, it is reasonable that we found a decreased alpha band power in random compared to blocked groups at the end of the practice period which increased from the practice to the retest condition for the random group but remained constant for the blocked group. These results show that the random practice effect is not specific to a distinct motor task. This is also supported in a study by Debarnot et al. (2015) showing a positive benefit of random practice in motor imagery. This is intriguing as different motor tasks lead to partly different cortical activation patterns but to the same behavioral effect, which is an increased performance after unstable practice. Therefore, it is more likely that the benefit of random practice reflects a general learning phenomenon.

However, there are several studies which did not observe a benefit of random practice and contradict the hypothesis of a general learning phenomenon (e.g., Brady, 1997; Jarus et al., 1997). This could be explained by the generally weak effect of random practice (Battig, 1979).

### Decreased Alpha Band Power in Parietal Electrodes Might Reveal Increased Parietal Processing

The EEG literature states that an increase of alpha band power reflects an active inhibition of the specific cortical region (Pfurtscheller, 2006; Klimesch, 2012) leading to weak contributions of this region to the current execution of a task. Therefore, the reverse of an increased alpha band power reflects a reduced inhibition of the region so that this region is contributing to the task execution. Using this hypothesis, our EEG data indicate that the parietal cortex is contributing to the motor adaptation task in random and blocked groups. However, the random group shows a slightly more decreased alpha band power at the end of the practice period which concurs with a previous study showing increased hemodynamic responses during interleaved practice (Lin et al., 2011). Despite the decreased alpha band power during practice, the random groups showed an increased alpha power in the retest condition. This observation indicates that the contribution of the parietal cortex decreases from the end of the practice period to the retest whereas it remains constant for the blocked group. This effect is somewhat contradictory to the behavioral data. Assuming a direct link between an increased task contribution of the parietal cortex with increased online feedback mechanisms would indicate that the random group performs better in the retest condition despite reduced online feedback mechanisms. As a direct link between alpha power of the parietal cortex and online feedback mechanisms is quite speculative, future work using neuroimaging techniques is needed.

### Parietal Alpha Band Power Is Negatively Correlated with the Motor Performance

The correlation coefficient show that our results in the alpha band power over contralateral and parietal electrodes are connected to the motor performance. Therefore, the observed effect in the alpha band does not reflect just a coincidence. The correlations were performed on differences between task periods. As all subjects increased their motor performance during the practice session, low differences between task periods reflect a more stabilized performance whereas high differences reflect performance loss. Therefore, the observed negative correlation coefficient indicates that a stabilized motor memory is accompanied by an increase of alpha band power from practice to retest. Independent correlation analyses for each group showed an even more decreased correlation coefficient for the random group compared to the blocked group. However, the reduction of the correlation coefficient accompanied with an increased *p*-value indicates that this test with only 12 subjects was underpowered.

However, it is not clear if the alpha band power of the parietal cortex is directly involved in the execution or correction of reaching movements or if it represents indirect influences which also could lead to the observed correlation.

### Limitations

The observed behavioral effects in the PDmax are only observed for the first trials of the retest period. Therefore, the positive effect of random practice in the dynamic adaptation task does not reflect a long term retest benefit. A specific warm-up could lead to similar performances between blocked and random practicing groups.

One could argue that the effect on the behavioral level is quite low because of the low *p*-value (*p* = 0.039). However, the effect size ($\eta_{p}^{2}$ = 0.18) is quite high which suggests that the amount of 24 subjects was too low and, thus, the study was underpowered.

Although the literature states the FFcomp as a measure of feedforward mechanisms, we cannot rule out that other control mechanisms slightly affected the results (impedance control, reflex modulations). Therefore, we cannot entirely rule out that random practice leads to a positive effect due to changes in impedance or feedforward control.

It is not clear if the effect on the behavioral level is facilitated by an increased memory retrieval due to random practice or by a decreased memory retrieval due to blocked practice. The latter could be explained by retroactive inhibition which might influence the retest performance of the blocked practicing subjects negatively. Retroactive inhibition describes the inhibition of memory by the acquisition of a new competing memory (Robertson et al., 2004). According to a blocked practice schedule, it is possible that the acquisition of a new task, or in our case of a new force field gradation, inhibits at least partially the previously learned task (Shewokis et al., 1998). From a theoretical point of view, if this is true, blocked practicing subjects will only be able to recall the last practiced task condition – the other task conditions will be inhibited (at least partially). This would lead to a decreased mean but increased variance in the retest motor performance of the blocked compared to the random group. As this phenomenon of retroactive inhibition is not restricted to dynamic motor adaptation tasks, it is not clear if previous work in the literature is also affected by retroactive inhibition. We are not able to rule this effect out and, therefore, this influence of retrograde inhibition should be carefully considered in future studies which try to compare blocked and random practice schedules.

## Conclusion

This work shows a positive effect of random practice in a dynamic motor adaptation task. Furthermore, this improved motor memory consolidation after random practice seems to be facilitated by mechanisms during movement execution and not by motor planning mechanisms. We assume that online feedback mechanisms during movement execution contribute to this phenomenon. The observed effects on the behavioral level are correlated with the alpha band power over parietal regions, suggesting that sensory processes play an important role. Altogether, this study indicates that the random practice effect reflects a task independent general learning phenomenon.

## Author Contributions

All authors (BT, CS, FP, TSc, and TSt) contributed to the conception and design of the study, drafting and critically revising of the work and the final approval of the version to be published. Data acquisition and analysis were done by BT and the interpretation of the data by BT, CS, and TSt.

## Conflict of Interest Statement

The authors declare that the research was conducted in the absence of any commercial or financial relationships that could be construed as a potential conflict of interest.

## References

[B1] Battig W. F. (1972). “Intratask interference as a source of facilitation in transfer and retention,” in *Topics in Learning and Performance* eds Thompson R. F. Voss J. F. (New York, NY: Academic Press) 131–159.

[B2] Battig W. F. (1979). “The flexibility of human memory,” in *Levels of Processing in Human Memory* eds Laird S. C. Fergus I. M. C. (Hillsdale, NJ: Lawrence Erlbaum Associates) 23–44.

[B3] Brady F. (1997). Contextual interference and teaching golf skills. *Percept. Mot. Skills* 48 347–350. 10.2466/pms.1997.84.1.347 9132730

[B4] Braun D. A. Aertsen A. Wolpert D. M. Mehring C. (2009). Learning optimal adaptation strategies in unpredictable motor tasks. *J. Neurosci.* 29 6472–6478. 10.1523/JNEUROSCI.3075-08.2009 19458218 PMC2692080

[B5] Canolty R. T. Edwards E. Dalal S. S. Soltani M. Nagarajan S. S. Kirsch H. E. (2006). High gamma power is phase-locked to theta oscillations in human neocortex. *Science* 313 1626–1628. 10.1126/science.1128115 16973878 PMC2628289

[B6] Cohen J. (1988). *Statistical Power Analysis for the Behavioral Sciences.* Hillsdale, NJ: Lawrence Erlbaum Associates.

[B7] Cohen M. X. (2014). *Analyzing Neural Time Series Data: Theory and Practice.* Cambridge, MA: MIT Press.

[B8] Cross E. S. Schmitt P. J. Grafton S. T. (2007). Neural substrates of contextual interference during motor learning support a model of active preparation. *J. Cogn. Neurosci.* 19 1854–1871. 10.1162/jocn.2007.19.11.1854 17958488

[B9] Debarnot U. Abichou K. Kalenzaga S. Sperduti M. Piolino P. (2015). Random motor imagery training induces sleep memory consolidation and transfer improvements. *Neurobiol. Learn. Mem.* 119 85–92. 10.1016/j.nlm.2014.12.010 25562401

[B10] Delorme A. Makeig S. (2004). EEGLAB: an open source toolbox for analysis of single-trial EEG dynamics. *J. Neurosci. Methods* 134 9–21. 10.1016/j.jneumeth.2003.10.009 15102499

[B11] Desmurget M. Epstein C. M. Turner R. S. Prablanc C. Alexander G. E. Grafton S. T. (1999). Role of the posterior parietal cortex in updating reaching movements to a visual target. *Nat. Neurosci.* 2 563–567. 10.1038/9219 10448222

[B12] Diedrichsen J. Hashambhoy Y. Rane T. Shadmehr R. (2005). Neural correlates of reach errors. *J. Neurosci.* 25 9919–9931. 10.1523/JNEUROSCI.1874-05.2005 16251440 PMC1479774

[B13] Dimitriou M. Wolpert D. M. Franklin D. W. (2013). The temporal evolution of feedback gains rapidly update to task demands. *J. Neurosci.* 33 10898–10909. 10.1523/JNEUROSCI.5669-12.2013 23804109 PMC3724995

[B14] Edgington E. S. Onghena P. (2007). *Randomization Tests.* London: CRC Press.

[B15] Franklin S. Wolpert D. M. Franklin D. W. (2012). Visuomotor feedback gains upregulate during the learning of novel dynamics. *J. Neurophysiol.* 108 467–478. 10.1152/jn.01123.2011 22539828 PMC3404796

[B16] Gardner E. P. Johnson K. O. (2013). “Sensory coding,” in *Principles of Neural Sciences* eds Kandel E. R. Schwartz J. H. Jessell T. M. Siegelbaum S. A. Hudspeth A. J. (New York, NY: McGraw-Hill) 449–474.

[B17] Gentili R. J. Bradberry T. J. Oh H. Hatfield B. D. Contreras Vidal J. L. (2011). Cerebral cortical dynamics during visuomotor transformation: adaptation to a cognitive-motor executive challenge. *Psychophysiology* 48 813–824. 10.1111/j.1469-8986.2010.01143.x 20964696

[B18] Hardwick R. M. Rottschy C. Miall R. C. Eickhoff S. B. (2013). A quantitative meta-analysis and review of motor learning in the human brain. *Neuroimage* 67 283–297. 10.1016/j.neuroimage.2012.11.020 23194819 PMC3555187

[B19] Huberdeau D. M. Krakauer J. W. Haith A. M. (2015). Dual-process decomposition in human sensorimotor adaptation. *Curr. Opin. Neurobiol.* 33 71–77. 10.1016/j.conb.2015.03.003 25827272

[B20] Jarus T. Wughalter E. H. Gianutsos J. G. (1997). Effects of contextual interference and conditions of movement task on acquisition, retention, and transfer of motor skills by women. *Percept. Mot. Skills* 84 179–193. 10.2466/pms.1997.84.1.179 9132708

[B21] Joiner W. M. Smith M. A. (2008). Long-term retention explained by a model of short-term learning in the adaptive control of reaching. *J. Neurophysiol.* 100 2948–2955. 10.1152/jn.90706.2008 18784273 PMC2585394

[B22] Kantak S. S. Sullivan K. J. Fisher B. E. Knowlton B. J. Winstein C. J. (2010). Neural substrates of motor memory consolidation depend on practice structure. *Nat. Neurosci.* 13 923–925. 10.1038/nn.2596 20622872

[B23] Kim S. Oh Y. Schweighofer N. (2015). Between-trial forgetting due to interference and time in motor adaptation. *PLoS ONE* 10:e0142963 10.1371/journal.pone.0142963 PMC4657926 26599075

[B24] Klimesch W. (2012). Alpha-band oscillations, attention, and controlled access to stored information. *Trends Cogn. Sci.* 16 606–617. 10.1016/j.tics.2012.10.007 23141428 PMC3507158

[B25] Lee T. D. Magill R. A. (1983). The locus of contextual interference in motor-skill acquisition. *J. Exp. Psychol. Learn. Mem. Cogn.* 9 730–746. 10.1037/0278-7393.9.4.730

[B26] Li Y. Wright D. L. (2000). An assessment of the attention demands during random- and blocked-practice schedule. *Q. J. Exp. Psychol. A* 53 591–606. 10.1080/713755890 10881620

[B27] Lin C. H. J. Chiang M. C. Knowlton B. J. Iacoboni M. Udompholkul P. Wu A. D. (2013). Interleaved practice enhances skill learning and the functional connectivity of fronto-parietal networks. *Hum. Brain Mapp.* 34 1542–1558. 10.1002/hbm.22009 22359276 PMC6870452

[B28] Lin C. H. J. Knowlton B. J. Chiang M. C. Iacoboni M. Udompholkul P. Wu A. D. (2011). Brain–behavior correlates of optimizing learning through interleaved practice. *Neuroimage* 56 1758–1772. 10.1016/j.neuroimage.2011.02.066 21376126

[B29] Magill R. A. Hall K. G. (1990). A review of the contextual interference effect in motor skill acquisition. *Hum. Mov. Sci.* 9 241–289. 10.1016/0167-9457(90)90005-X

[B30] Makeig S. Bell A. J. Jung T. P. Sejnowski T. J. (1996). “Independent component analysis of electroencephalographic data,” in *Advances in Neural Information Processing Systems* eds Touretzky D. Mozer M. Hasselmo M. (Cambridge, MA: MIT Press) 145–151.

[B31] Oldfield R. C. (1971). The assessment and analysis of handedness: the Edinburgh inventory. *Neuropsychologia* 9 97–113. 10.1016/0028-3932(71)90067-4 5146491

[B32] Pascual-Leone A. Wassermann E. M. Grafman J. Hallett M. (1996). The role of the dorsolateral pre-frontal cortex in implicit procedural learning. *Exp. Brain Res.* 107 479–485. 10.1007/BF00230427 8821387

[B33] Perfetti B. Moisello C. Landsness E. C. Kvint S. Lanzafame S. Onofrj M. (2011). Modulation of gamma and theta spectral amplitude and phase synchronization is associated with the development of visuo-motor learning. *J. Neurosci.* 31 14810–14819. 10.1523/JNEUROSCI.1319-11.2011 21994398 PMC3206224

[B34] Pfurtscheller G. (2006). “The cortical activation model (CAM),” in *Progress in Brain Research. Event-Related Dynamics of Brain Oscillations* eds Neuper C. Klimesch W. (Amsterdam: Elsevier) 19–27. 10.1016/S0079-6123(06)59002-8 17071221

[B35] Pfurtscheller G. Lopes da Silva F. H. (1999). Event-related EEG/MEG synchronization and desynchronization: basic principles. *Clin. Neurophysiol.* 110 1842–1857. 10.1016/S1388-2457(99)00141-8 10576479

[B36] Rathelot J.-A. Dum R. P. Strick P. L. (2017). Posterior parietal cortex contains a command apparatus for hand movements. *Proc. Natl. Acad. Sci. U.S.A.* 114 4255–4260. 10.1073/pnas.1608132114 28373554 PMC5402465

[B37] Richardson J. T. E. (2011). Eta squared and partial eta squared as measures of effect size in educational research. *Educ. Res. Rev.* 6 135–147. 10.1016/j.edurev.2010.12.001

[B38] Robertson E. M. (2007). The serial reaction time task: implicit motor skill learning? *J. Neurosci.* 27 10073–10075. 10.1523/JNEUROSCI.2747-07.2007 17881512 PMC6672677

[B39] Robertson E. M. (2009). From creation to consolidation: a novel framework for memory processing. *PLoS Biol.* 7:e1000019 10.1371/journal.pbio.1000019 PMC2631067 19175290

[B40] Robertson E. M. Pascual-Leone A. Miall R. C. (2004). Current concepts in procedural consolidation. *Nat. Rev. Neurosci.* 5 576–582. 10.1038/nrn1426 15208699

[B41] Roux F. Uhlhaas P. J. (2014). Working memory and neural oscillations: alpha-gamma versus theta-gamma codes for distinct WM information? *Trends Cogn. Sci.* 18 16–25. 10.1016/j.tics.2013.10.010 24268290

[B42] Scheidt R. A. Dingwell J. B. Mussa-Ivaldi F. A. (2001). Learning to move amid uncertainty. *J. Neurophysiol.* 86 971–985. 11495965 10.1152/jn.2001.86.2.971

[B43] Scheidt R. A. Reinkensmeyer D. J. Conditt M. A. Rymer W. Z. Mussa-Ivaldi F. A. (2000). Persistence of motor adaptation during constrained, multi-joint, arm movements. *J. Neurophysiol.* 84 853–862. 10938312 10.1152/jn.2000.84.2.853

[B44] Schlögl A. Keinrath C. Zimmermann D. Scherer R. Leeb R. Pfurtscheller G. (2007). A fully automated correction method of EOG artifacts in EEG recordings. *Clin. Neurophysiol.* 118 98–104. 10.1016/j.clinph.2006.09.003 17088100

[B45] Schwarb H. Schumacher E. H. (2009). Neural evidence of a role for spatial response selection in the learning of spatial sequences. *Brain Res.* 1247 114–125. 10.1016/j.brainres.2008.09.097 18976640

[B46] Shadmehr R. Brandt J. Corkin S. (1998). Time-dependent motor memory processes in amnesic subjects. *J. Neurophysiol.* 80 1590–1597. 9744966 10.1152/jn.1998.80.3.1590

[B47] Shadmehr R. Krakauer J. W. (2008). A computational neuroanatomy for motor control. *Exp. Brain Res.* 185 359–381. 10.1007/s00221-008-1280-5 18251019 PMC2553854

[B48] Shadmehr R. Mussa-Ivaldi F. A. (1994). Adaptive representation of dynamics during learning of a motor task. *J. Neurosci.* 14 3208–3224. 8182467 10.1523/JNEUROSCI.14-05-03208.1994 PMC6577492

[B49] Shea J. B. Morgan R. L. (1979). Contextual interference effects on the acquisition, retention, and transfer of a motor skill. *J. Exp. Psychol. Hum. Learn.* 5 179–187. 10.1037/0278-7393.5.2.179

[B50] Shewokis P. A. Del Rey P. Simpson K. J. (1998). A test of retroactive inhibition as an explanation of contextual interference. *Res. Q. Exerc. Sport* 69 70–74. 10.1080/02701367.1998.10607669 9532625

[B51] Stockinger C. Focke A. Stein T. (2014). Catch trials in force field learning influence adaptation and consolidation of human motor memory. *Front. Hum. Neurosci.* 8:231 10.3389/fnhum.2014.00231 PMC4001009 24795598

[B52] Stockinger C. Pöschl M. Focke A. Stein T. (2012). ManipAnalysis - a software application for the analysis of force field experiments. *Int. J. Comput. Sci. Sport* 11 52–57.

[B53] Stockinger C. Thürer B. Focke A. Stein T. (2015). Intermanual transfer characteristics of dynamic learning: direction, coordinate frame, and consolidation of interlimb generalization. *J. Neurophysiol.* 114 3166–3176. 10.1152/jn.00727.2015 26424581 PMC4686290

[B54] Tanaka S. Honda M. Hanakawa T. Cohen L. G. (2010). Differential contribution of the supplementary motor area to stabilization of a procedural motor skill acquired through different practice schedules. *Cereb. Cortex* 20 2114–2121. 10.1093/cercor/bhp276 20038545 PMC2923213

[B55] Taylor J. A. Krakauer J. W. Ivry R. B. (2014). Explicit and implicit contributions to learning in a sensorimotor adaptation task. *J. Neurosci.* 34 3023–3032. 10.1523/JNEUROSCI.3619-13.2014 24553942 PMC3931506

[B56] Thürer B. Stockinger C. Focke A. Putze F. Schultz T. Stein T. (2016). Increased gamma band power during movement planning coincides with motor memory retrieval. *Neuroimage* 125 172–181. 10.1016/j.neuroimage.2015.10.008 26458517

[B57] Tombini M. Zappasodi F. Zollo L. Pellegrino G. Cavallo G. Tecchio F. (2009). Brain activity preceding a 2D manual catching task. *Neuroimage* 47 1735–1746. 10.1016/j.neuroimage.2009.04.046 19389476

[B58] Wright D. Verwey W. Buchanen J. Chen J. Rhee J. Immink M. (2015). Consolidating behavioral and neurophysiologic findings to explain the influence of contextual interference during motor sequence learning. *Psychon. Bull. Rev.* 23 1–21. 10.3758/s13423-015-0887-3 26084879

[B59] Yousif N. Diedrichsen J. (2012). Structural learning in feedforward and feedback control. *J. Neurophysiol.* 108 2373–2382. 10.1152/jn.00315.2012 22896725 PMC3545174

